# Three-Dimensional Quantitative Evaluation of the Scapular Skin Marker Movements in the Upright Posture

**DOI:** 10.3390/s22176502

**Published:** 2022-08-29

**Authors:** Yuki Yoshida, Noboru Matsumura, Yoshitake Yamada, Minoru Yamada, Yoichi Yokoyama, Azusa Miyamoto, Masaya Nakamura, Takeo Nagura, Masahiro Jinzaki

**Affiliations:** 1 Department of Orthopedic Surgery, Keio University School of Medicine, 35 Shinanomachi, Shinjuku-ku, Tokyo 160-8582, Japan; 2 Department of Radiology, Keio University School of Medicine, 35 Shinanomachi, Shinjuku-ku, Tokyo 160-8582, Japan

**Keywords:** upright computed tomography, marker displacement, soft tissue artifact, shoulder, optical motion capture systems

## Abstract

Motion capture systems using skin markers are widely used to evaluate scapular kinematics. However, soft-tissue artifact (STA) is a major limitation, and there is insufficient knowledge of the marker movements from the original locations. This study explores a scapular STA, including marker movements with shoulder elevation using upright computed tomography (CT). Ten healthy males (twenty shoulders in total) had markers attached to scapular bony landmarks and underwent upright CT in the reference and elevated positions. Marker movements were calculated and compared between markers. The bone-based and marker-based scapulothoracic rotation angles were also compared in both positions. The median marker movement distances were 30.4 mm for the acromial angle, 53.1 mm for the root of the scapular spine, and 70.0 mm for the inferior angle. Marker movements were significantly smaller on the superolateral aspect of the scapula, and superior movement was largest in the directional movement. Scapulothoracic rotation angles were significantly smaller in the marker-based rotation angles than in the bone-based rotation angles of the elevated position. We noted that the markers especially did not track the inferior movement of the scapular motion with shoulder elevation, resulting in an underestimation of the marker-based rotation angles.

## 1. Introduction

Analysis of skeletal kinematics has improved our knowledge of human function and is required in different clinical and sports applications. Although various devices have been utilized to evaluate skeletal kinematics, a medical image analysis using magnetic resonance imaging (MRI) [1], computed tomography (CT) [2], or fluoroscopy [3] has a narrow field of view and low time resolution, which limits its use for analyzing wide or fast movements. A bone–pin analysis [4] that can be accurately assessed has the problem of a high invasiveness. Because of these limitations, motion capture systems using skin markers are widely used for non-invasive motion analysis [5].

In the analysis of scapula kinematics using optical motion capture systems, a major problem is that the soft-tissue artifact (STA) is larger than that of other bones [6] and there is a high propensity for error, owing to the deep location and gliding nature beneath the skin surface slipping between the skin and bone [7]. To reduce this error, several studies evaluated scapular STA using palpation, bone–pin, fluoroscopy, or bi-plane radiography [8,9,10,11,12,13,14], and compensation methods for the scapular STA, such as the acromion marker cluster method [15] and multibody kinematics optimization [16,17] were developed. There have been many comparative validation studies on scapular rotation angles [8,9,10,11,12,13,14], but the marker movements themselves have seldom been evaluated, and there is insufficient knowledge of marker movements from the original locations.

There are few studies that directly evaluated marker movements of scapula STA using MRI in the supine position [7,18]. Although shoulder kinematics are generally evaluated in the upright posture [19,20,21], no study has evaluated scapular STA, including three-dimensional (3D) marker movements in the upright position using CT scans, which are widely used to detect bone conditions. For an accurate analysis, it is necessary to quantitatively evaluate how the scapular skin markers move with shoulder elevation from the original locations, and this should be confirmed in the upright posture.

Recently, upright CT scanners have been developed that have physical characteristics comparable to those of conventional CT machines, which enables the capturing of 3D marker movements in the upright position [22]. The visualization and evaluation of the actual 3D marker movements at the bony landmarks of the scapula coordinate system may provide important clues regarding scapular STA compensations. The purpose of this study was to visualize the 3D scapular skin marker movements with shoulder elevation in the upright position and to clarify the scapular STA by evaluating the movement distance of each scapular skin marker and the differences between bone-based and marker-based scapulothoracic rotation angles. 

## 2. Materials and Methods

### 2.1. Participants

**Figure 1:**
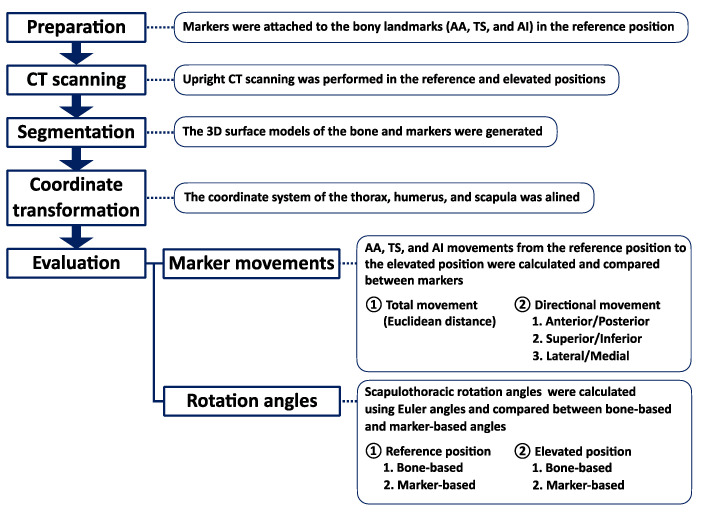
Flowchart of this study. CT, computed tomography; AA, acromial angle (angulus acromialis); TS, root of the scapular spine (trigonum spinae scapulae); AI, inferior angle (angulus inferior).

This study was approved by our Institutional Review Board (study protocol: #20150293) and included ten male participants with twenty healthy shoulders who had no history of injury or surgery. All participants were right-arm-dominant. The mean age, height, body weight, and body mass index (BMI) were 21.6 ± 1.6 years, 169.6 ± 5.4 cm, 63.7 ± 3.4 kg, and 22.2 ± 1.4 kg/m^2^ respectively. All participants provided informed consent, agreed to participate in this study, and underwent upright CT (prototype TSX-401R; Canon Medical Systems Corporation, Otawara, Japan) [23,24]. The flowchart of this study is shown in Figure 1.

### 2.2. Image Acquisition

**Figure 2:**
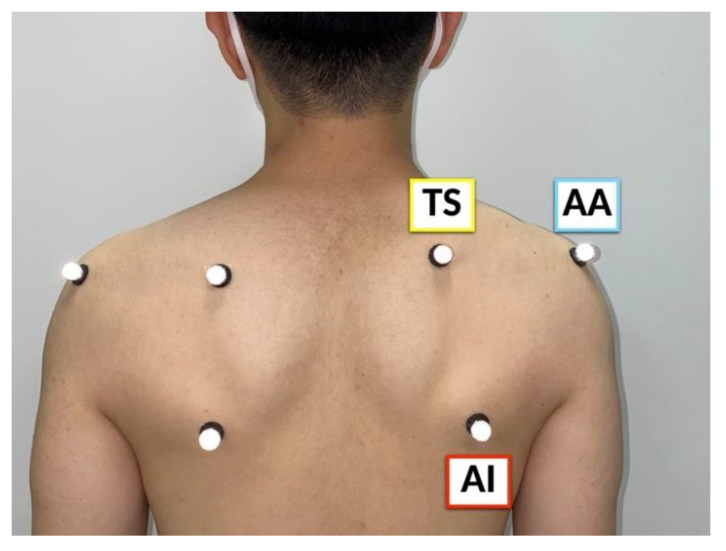
Skin markers were attached to the acromial angle (angulus acromialis [AA]), root of the scapular spine (trigonum spinae scapulae [TS]), and inferior angle (angulus inferior [AI]) of the participants.

**Figure 3:**
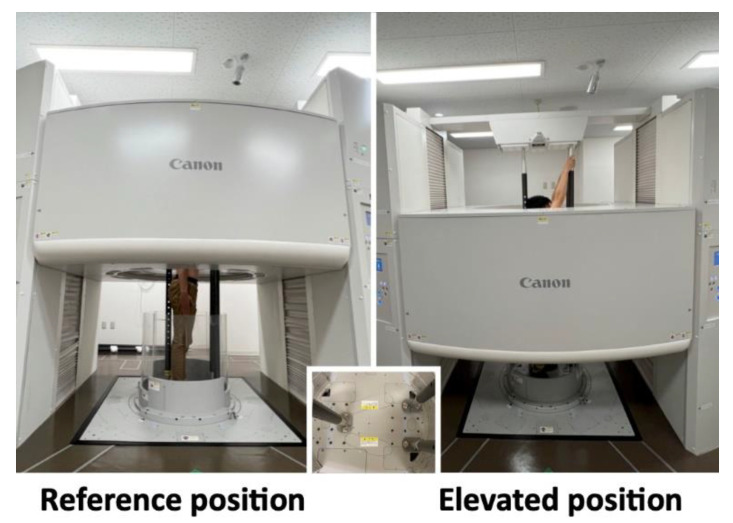
Upright computed tomography. To keep the reference and elevated positions during the scanning, three vertical poles were installed in the gantry.

Spherical skin markers were attached to participants’ scapular bony landmarks with their arms by their sides according to the International Society of Biomechanics (ISB) recommendation [25]. The bony landmarks of the scapula were the acromial angle (angulus acromialis [AA]), root of the scapular spine (trigonum spinae scapulae [TS]), and inferior angle (angulus inferior [AI]) (Figure 2). They were instructed to stand in the upright CT gantry, and scanning was performed in the reference and elevated positions. To keep these positions and avoid motion artifacts during scanning, three vertical poles were installed in the upright CT gantry (Figure 3). In the reference position, the upper extremities were positioned with the palms touching the lateral sides. In the elevated position, the upper extremities were elevated to the maximum, and the participants’ hands were placed on the poles in front of them. Scanning was performed at 120 kVp and at a gantry rotation speed of 0.5 s in the helical scan mode (80-detector row), with a noise index of 24 and helical pitch of 0.8 for the body trunk. Image reconstruction was performed using Adaptive Iterative Dose Reduction 3D (Canon Medical Systems Corporation, Otawara, Japan), which minimizes imaging radiation [26]. CT data were accumulated in Digital Imaging and Communication in Medicine (DICOM) data format.

### 2.3. Coordinate Systems

**Figure 4:**
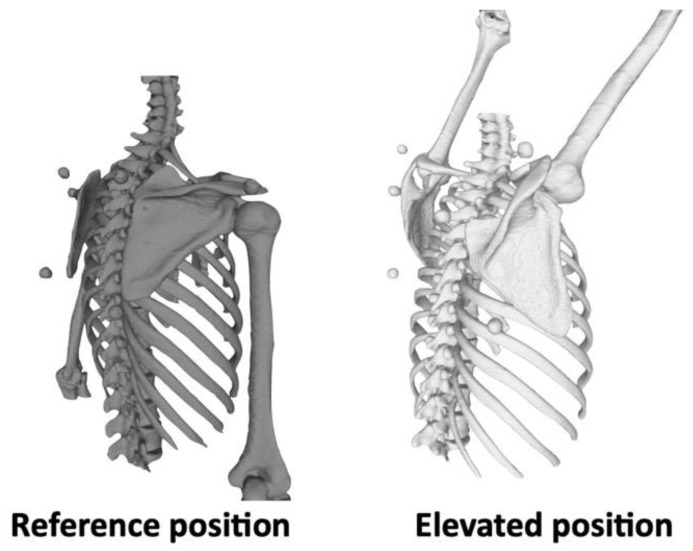
Three-dimensional surface models in the reference position (gray) and elevated position (white).

The 3D surface models of the bone and markers were segmented from the DICOM data using Avizo software (version 9.3.0; Thermo Fisher Scientific, Tokyo, Japan). Segmentation was manually performed to identify the bone and markers in each slice of multiplanar reformatting at both the reference and elevated positions and 3D surface models were exported as Standard Triangulated Language (STL) data [27] (Figure 4). 

The bony landmarks were identified on STL data of 3D bone surface models, and the coordinate systems of the thorax, humerus, and scapula were defined according to ISB recommendations [25] using MeshLab software (version 1.3.3; Institute of Information Science and Technologies, Pisa, Italy). 

The thoracic coordinate system was defined as follows: The origin was coincident with the deepest point of the sternal notch. The Y-axis was defined as the line connecting the midpoint between the xiphoid process and the spinal process of the eighth thoracic vertebra and the midpoint between the sternal notch and spinal process of the seventh cervical vertebra, pointing upward. The Z-axis was the line perpendicular to the plane formed by the sternal notch, seventh cervical vertebra, and midpoint between xiphoid process and eighth thoracic vertebra, pointing to the right. The X-axis was the common line perpendicular to the Z- and Y-axes, pointing forward.

The humerus coordinate system was defined as follows: The origin was coincident with center of the humeral head using a sphere fit based on the convex articulating surface above the anatomical neck [28]. The Y-axis was defined as the line connecting the center of the humeral head and the midpoint of the most caudal point on the lateral and medial epicondyles, proximally pointing to center of the humeral head. The X-axis was the line perpendicular to the plane formed by the lateral and medial epicondyles and the center of the humeral head, pointing forward. The Z-axis was the common line perpendicular to the X- and Y-axes, pointing to the right.

**Figure 5:**
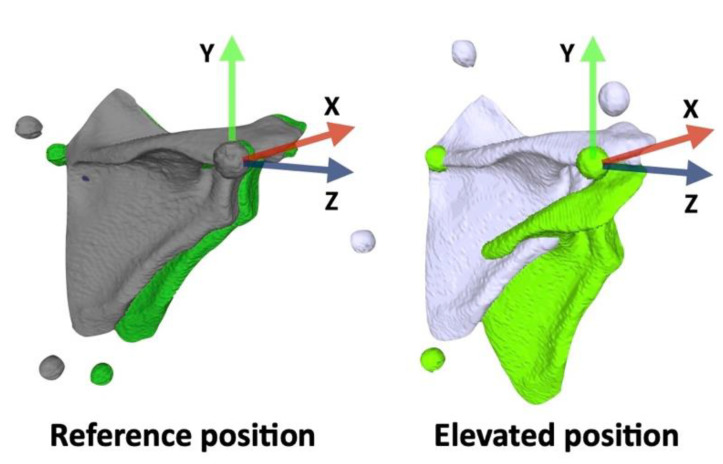
The scapular rotation was evaluated by bone-based (gray and white) and marker-based (dark green and light green) scapula coordinate systems.

The scapular coordinate system was defined as follows: The origin was coincident with AA. The Z-axis was defined as the line connecting TS and AA, pointing to the right. The X-axis was the line perpendicular to the plane formed by AI, AA, and TS, pointing forward. The Y-axis was the common line perpendicular to the X- and Z-axes, pointing upward. The scapular rotation was evaluated by both bone-based and marker-based scapula coordinate systems (Figure 5).

### 2.4. Marker Movements

**Figure 6:**
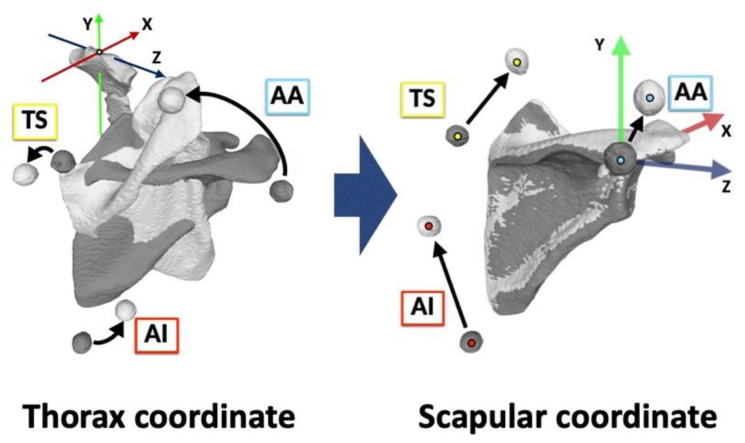
Visualization of the scapular skin marker movements. Black arrows show the marker movements from the reference position (gray) to the elevated position (white). The 3D movements of the scapula and markers were visualized on the thorax coordinate system. The 3D scapular marker displacement was identified aligned to the scapular coordinate system. AA, acromial angle (angulus acromialis); TS, root of the scapular spine (trigonum spinae scapulae); AI, inferior angle (angulus inferior).

Marker movements were visualized by matching the scapula in the reference and elevated positions (Figure 6). The 3D locations of each marker were identified on the bone-based scapular coordinate system by calculating the center of the markers. 

The total marker movement distances of AA, TS, and AI from the reference position to the elevated position were obtained using the center translation distances of these markers by calculating Euclidean distance.

$$Total\;movement\;distance=\sqrt{{\left(X_{e}-X_{r}\right)}_{2}+{\left(Y_{e}-Y_{r}\right)}_{2}+{\left(Z_{e}-Z_{r}\right)}_{2}}\;$$

where e—elevated position; r—reference position.

**Figure 7:**
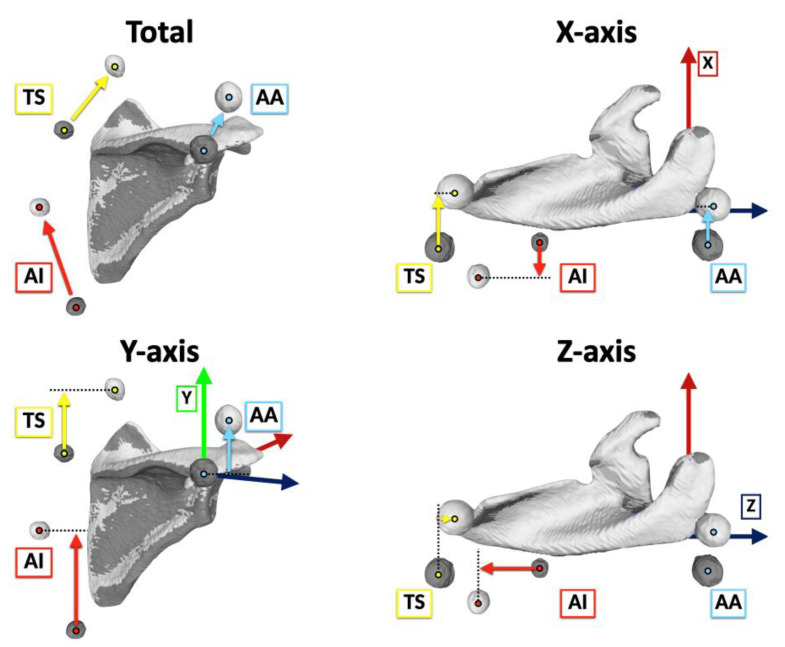
Total and directional marker movements from the reference position (gray) to the elevated position (white). The directional movement was described as anterior/posterior movement of the X-axis translation, superior/inferior movement of the Y-axis translation, and lateral/medial movement of the Z-axis translation. Light blue arrows, yellow arrows, and red arrows indicate the acromial angle (angulus acromialis [AA]), root of the scapular spine (trigonum spinae scapulae [TS]), and inferior angle (angulus inferior [AI]) marker movements, respectively.

The directional movement was described as the anterior/posterior movement of the X-axis translation, superior/inferior movement of the Y-axis translation, and lateral/medial movement of the Z-axis translation [29] (Figure 7).

$$Anterior\;movement\;distance=X_{e}-X_{r}$$

$$Superior\;movement\;distance=Y_{e}-Y_{r}$$

$$Lateral\;movement\;distance=Z_{e}-Z_{r}$$

where e—elevated position; r—reference position.

### 2.5. Rotation Angles

The angular rotations of the humerus and scapula were calculated using Euler angles relative to the thorax following the ISB recommendations [25]. The humerothoracic elevation angles in the reference and elevated positions were calculated using the X-axis of the Y-X-Y sequence. The scapulothoracic rotation angles of both bone-based and marker-based scapulae in the reference and elevated positions were calculated using Y-X-Z sequence. The scapular rotation was described as the upward/downward rotation in the X-axis, internal/external rotation in the Y-axis, and anterior/posterior tilting in the Z-axis.

### 2.6. Statistical Analysis

SPSS Statistics 27.0.1.0 software (IBM Corp., Armonk, NY, USA) was used for the statistical analyses. The intra- and inter-observer reliabilities of the marker movement distances were assessed by calculating intraclass correlation coefficient (ICC) based on 10 randomly selected markers. The measurements were blinded to the two authors (ICC model 2,1) and repeated by one author with a 6-month interval (ICC model 1,1). After the reliabilities were determined to be acceptable, the marker movement distances for all 60 markers from the 20 shoulders were assessed by one author.

Descriptive statistics of the marker movement distances were presented using median and interquartile range (IQR) as the non-normal distribution (Shapiro–Wilk normality test, *p* < 0.05). The differences in marker movement distances were evaluated using Friedman tests, followed by Wilcoxon tests with Bonferroni adjustments. 

Descriptive statistics of the rotation angles were presented using mean and standard as normal distribution (Shapiro–Wilk normality test, *p* ≥ 0.05). The differences in scapulothoracic rotation angles between the bone-based and marker-based scapulae at each position were evaluated using paired *t*-tests. 

The correlation between the marker movement distances, and the differences between the bone-based and marker-based scapulothoracic rotation angles in the elevated position, was evaluated using Spearman’s rank correlation analysis. The correlation between the marker movement distances and subjects’ BMI was also evaluated using Spearman’s rank correlation analysis. The significance level was set at 0.05 for all analyses.

## 3. Results

The intra- and inter-observer correlation coefficients for the total marker movement distances were 0.963 (95% confidence interval [CI], 0.856–0.991) and 0.952 (95% CI, 0.811–0.989), respectively. Those for directional marker movement distances were 0.994 (95% CI, 0.975–0.999) and 0.994 (95% CI, 0.976–0.999) for X-axis translation, 0.966 (95% CI, 0.867–0.992) and 0.957 (95% CI, 0.830–0.990) for Y-axis translation, and 0.978 (95% CI, 0.914–0.995) and 0.967 (95% CI, 0.870–0.992) for Z-axis translation, respectively. These results confirmed that the data are highly reproducible.

**Figure 8:**
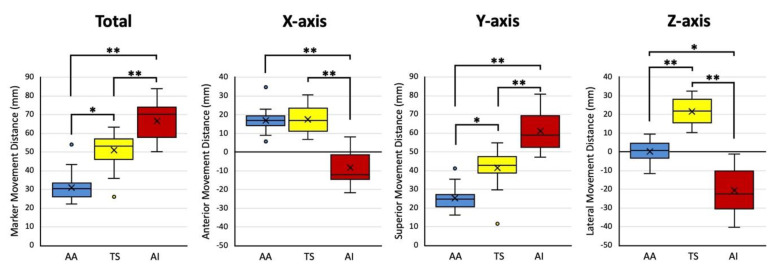
Boxplots of total, X-axis (anterior), Y-axis (superior), and Z-axis (lateral) marker movement distances of the acromial angle (angulus acromialis [AA]), root of the scapular spine (trigonum spinae scapulae [TS]), and inferior angle (angulus inferior [AI]). Box and whiskers quantify median, quartile, and extreme values. Outliers are shown with a black circle. “×” marks mean values. * *p* < 0.05; ** *p* < 0.01.

The median marker movement distances were 30.4 (IQR 25.9–33.2) mm for AA, 53.1 (IQR 46.1–56.8) mm for TS, and 70.0 (IQR 57.8–73.9) mm for AI. It was significantly smaller on the superolateral aspect of the scapula and significantly larger on the inferomedial aspect of the scapula. In the X-axis translation, the median anterior movement distances were 16.7 (IQR 14.0–19.3) mm for AA and 16.7 (IQR 10.9–23.2) mm for TS. The median posterior movement distance was 12.1 (IQR 1.5–14.6) mm for AI. In the Y-axis translation, the median superior movement distances were 24.5 (IQR 20.6–27.2) mm for AA, 42.7 (IQR 38.5–47.4) mm for TS, and 58.7 (IQR 52.3–69.2) mm for AI. In the Z-axis translation, the median lateral movement distances were 0.7 (IQR −3.5–4.4) mm for AA and 21.6 (IQR 15.3–27.9) mm for TS. The median medial movement distance was 22.5 (IQR 10.3–30.6) mm for AI. In the directional movement, the superior movements were the largest for all markers (Figure 8).

The mean humerothoracic elevation angles were 4.3° ± 2.2° in the reference position and 146.1° ± 4.5° in the elevated position. 

**Figure 9:**
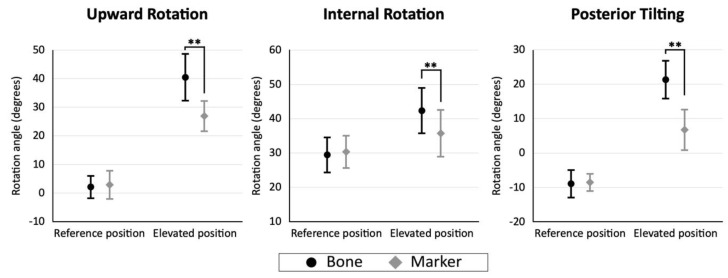
Bone-based (black circles) and marker-based (gray rhombus) scapulothoracic rotation angles in the reference and the elevated positions. The error bars indicate the standard deviation. ** *p* < 0.01.

In the reference position, the mean bone-based and marker-based scapulothoracic rotation was 2.2° ± 4.2° and 2.9° ± 5.3° in upward rotation, 29.5° ± 5.4° and 30.4° ± 4.9° in internal rotation, and 8.8° ± 4.2° and 8.5° ± 2.7° in anterior tilting. No difference was found between bone-based and marker-based rotations (*p* = 0.356, 0.335, and 0.737, respectively). In the elevated position, the mean bone-based and marker-based scapulothoracic rotation was 40.5° ± 8.5° and 27.0° ± 5.6° in upward rotation, 42.3° ± 6.8° and 35.8° ± 7.0° in the internal rotation, and 21.3° ± 5.8° and 6.8° ± 6.1° in posterior tilting. The scapulothoracic rotation angles were significantly smaller in the marker-based rotation angles than in the bone-based rotation angles (*p* < 0.001, *p* = 0.003, and *p* < 0.001, respectively) (Figure 9). The mean differences between the bone-based and marker-based scapulothoracic rotation angles were 13.5° ± 5.9° in upward rotation, 6.6° ± 8.3° in internal rotation, and 14.6° ± 7.2° in posterior tilting.

In the correlation analysis between the marker movement distances and the differences in scapulothoracic rotation angles, positive correlations were observed between AA movement distances and posterior tilting, between TS movement distances and internal rotation, and between AI movement distances and the upward rotation (Table 1). Regarding the correlation between marker movement distances and BMI, AA showed a strong positive correlation with BMI, whereas TS and AI showed no correlation with BMI (AA, ρ = 0.713, *p* < 0.001; TS, ρ = −0.216, *p* = 0.361; AI, ρ = −0.326, *p* < 0.161).

## 4. Discussion

This study provided quantitative data on the 3D scapular skin marker movements caused by shoulder elevation in the upright position. The differences between bone-based and marker-based scapulothoracic rotation angles in the reference and elevated positions were also clarified with no compensation of the marker displacements. Upright CT allows for an evaluation of the same position as the general scapular motion analysis and a visualization of the 3D marker movements. The marker movement distance was the smallest in AA, and superior movement was largest in the directional movement. Additionally, this study evaluated the correlations between the marker movements and the scapular STA of the rotation angles. The marker movements of AA, TS, and AI were related to differences in scapular posterior tilting, internal rotation, and upward rotation, respectively.

The results of the total marker movement distances for the medial and lateral aspects of the scapula were similar to those obtained in the supine position described by a previous study [7], with a larger marker displacement in the medial aspect than in the lateral aspect. Conversely, the results of the superomedial and inferomedial aspects of the scapula were different. The marker displacement was significantly smaller on the superomedial aspect than in the inferomedial aspect in the upright position, while no difference was observed in the previous supine study. This might be caused by scapula alignment changes in the upright and supine positions [30] and skin tension changes due to gravity.

As for the directional movement, a previous study evaluating directional bias in the supine position [18] reported that the superior movement was the largest and most consistent with this study. Superior movement might occur for the following reasons: TS and AI could not track the inferior movement of the medial aspect of the scapula associated with upward rotation, and AA could not track the inferior movement of the acromion associated with posterior tilt. Thus, the markers superiorly moved from the original locations, and this resulted in an underestimation of the scapulothoracic rotation angles in the elevated position. This suggests that superior movement is especially responsible for scapular STA during elevation and requires the most modifications. 

The present results indicate that the AA movement was smallest, which supports the acromion marker cluster method, where a cluster of markers is placed on the acromion to minimize STA [15]. However, AA had a strong positive correlation with BMI, even with the small variation in the physique of our study group. A larger physique might be more influenced by the deltoid muscle contraction and AA should be attached to a less susceptible region, such as the meeting point between the acromion and the scapular spine [31]. Otherwise, it would be preferable to use multiple calibrations [20,32] or multibody kinematic optimization [16,17] to reduce STA. Moreover, various optimization techniques have recently been developed in other fields, and it is hoped that more actual scapular tracking methods will be invented to apply these techniques [33,34,35,36].

The strength of this study was the direct visualization of the STA of scapular markers using a newly developed upright CT scanner. On the other hand, there are several limitations of this study. The first limitation is the small number of cases examined: twenty shoulders (ten participants), and physique differences were not evaluated. It is known that obese individuals have larger STA in the lower extremities [37,38]. Although only AA movement distance was correlated with BMI in this study, the other marker distances could also be correlated with BMI if we evaluated obese individuals. The second limitation was the lack of evaluation of female and older individuals. Differences in scapular alignment between males and females [30], skeletal alignment [39], and skin surface [40] changes with age have been reported, and skin motion artifacts might be different. The third limitation was that marker movements were evaluated for only three bony landmarks of the scapula. The STA of the other points on the scapula and other bones was not evaluated. The fourth limitation was that marker movements were only evaluated with shoulder elevation, and other shoulder motions were not evaluated. Further studies are needed to more fully understand the STA, such as evaluating different individuals, points, and motions. Even with these limitations, this study clarified the details of the 3D scapular skin marker movements, and our results will be helpful for understanding scapular STA with shoulder elevation. 

## 5. Conclusions

This study clarified the scapular STA with the quantitative evaluation of the marker movement using an upright CT scanner. We noted that the markers did not track, in particular, the inferior movement of the scapular motion with shoulder elevation, therefore resulting in an underestimation of the marker-based scapulothoracic rotation angles. The marker movement was larger on the inferomedial aspect of the scapula and smaller on the superolateral aspect of the scapula, and superior movement was largest in the directional movement. These results will be helpful for understanding scapular STA with shoulder elevation.

## Figures and Tables

**Table 1:** The correlation between the marker movement distances and the differences between the bone-based and marker-based scapulothoracic rotation angles in the elevated position. * *p* < 0.05.

	Spearman’s Rank Correlation Coefficient	Upward Rotation	Internal Rotation	Posterior Tilting
Acromial angle (angulus acromialis [AA])	ρ (*p*-value)	0.281 (0.231)	0.017 (0.945)	0.535 (0.015 *)
Root of the scapular spine (trigonum spinae scapulae [TS])	ρ (*p*-value)	0.350 (0.131)	0.449 (0.047 *)	0.184 (0.439)
Inferior angle (angulus inferior [AI])	ρ (*p*-value)	0.453 (0.045 *)	0.280 (0.232)	0.117 (0.622)

## Data Availability

The datasets used and/or analyzed during the study are available from the corresponding author on reasonable request.

## Abbreviations

AA	acromial angle (angulus acromialis);
AI	inferior angle (angulus inferior)
BMI	body mass index
CI	confidence interval
CT	computed tomography
DICOM	Digital Imaging and Communication in Medicine
ICC	intraclass correlation coefficient
IQR	interquartile range
ISB	International Society of Biomechanics
MRI	magnetic resonance imaging
STL	Standard Triangulated Language
STA	soft-tissue artifact
TS	root of the scapular spine (trigonum spinae scapulae)
3D	three-dimensional
